# Thiopurines impair the apical plasma membrane expression of CFTR in pancreatic ductal cells via RAC1 inhibition

**DOI:** 10.1007/s00018-022-04662-y

**Published:** 2023-01-07

**Authors:** Bálint Tél, Noémi Papp, Árpád Varga, Viktória Szabó, Marietta Görög, Petra Susánszki, Tim Crul, Aletta Kis, Ingrid H. Sendstad, Mária Bagyánszki, Nikolett Bódi, Péter Hegyi, József Maléth, Petra Pallagi

**Affiliations:** 1 Department of Pediatrics, Semmelweis University, Budapest, Hungary; 2 Department of Medicine, University of Szeged, Szeged, Hungary; 3 HCEMM-USZ Molecular Gastroenterology Research Group, University of Szeged, Szeged, Hungary; 4 ELKH-USZ Momentum Epithelial Cell Signaling and Secretion Research Group, Department of Medicine, University of Szeged, Szeged, Hungary; 5 Department of Physiology, Anatomy, and Neuroscience, Faculty of Science and Informatics, University of Szeged, Szeged, Hungary; 6 Centre for Translational Medicine and Division for Pancreatic Disorder, Semmelweis University, Budapest, Hungary; 7 Institute for Translational Medicine, University of Pécs, Pécs, Hungary

**Keywords:** Thiopurine-induced pancreatitis, Drug-induced pancreatitis, Pancreatic ductal secretion, Azathioprine, Murine pancreas

## Abstract

**Background and aims:**

Thiopurine-induced acute pancreatitis (TIP) is one of the most common adverse events among inflammatory bowel disease patients treated with azathioprine (AZA), representing a significant clinical burden. Previous studies focused on immune-mediated processes, however, the exact pathomechanism of TIP is essentially unclear.

**Methods:**

To model TIP in vivo, we triggered cerulein-induced experimental pancreatitis in mice receiving a daily oral dose of 1.5 mg/kg AZA. Also, freshly isolated mouse pancreatic cells were exposed to AZA ex vivo*,* and acinar cell viability, ductal and acinar Ca^2+^ signaling, ductal Cl^–^ and HCO_3_^–^ secretion, as well as cystic fibrosis transmembrane conductance regulator (CFTR) expression were assessed using microscopy techniques. Ras-related C3 botulinum toxin substrate (RAC1) activity was measured with a G-LISA assay. Super-resolution microscopy was used to determine protein colocalization.

**Results:**

We demonstrated that AZA treatment increases tissue damage in the early phase of cerulein-induced pancreatitis in vivo. Also, both *per os* and ex vivo AZA exposure impaired pancreatic fluid and ductal HCO_3_^–^ and Cl^–^ secretion, but did not affect acinar cells. Furthermore, ex vivo AZA exposure also inhibited RAC1 activity in ductal cells leading to decreased co-localization of CFTR and the anchor protein ezrin, resulting in impaired plasma membrane localization of CFTR.

**Conclusions:**

AZA impaired the ductal HCO_3_^–^ and Cl^–^ secretion through the inhibition of RAC1 activity leading to diminished ezrin-CFTR interaction and disturbed apical plasma membrane expression of CFTR. We report a novel direct toxic effect of AZA on pancreatic ductal cells and suggest that the restoration of ductal function might help to prevent TIP in the future.

**Supplementary Information:**

The online version contains supplementary material available at 10.1007/s00018-022-04662-y.

## Introduction

Acute pancreatitis (AP) is one of the most frequent causes of hospitalization among non-malignant gastrointestinal diseases [1], moreover, the mortality of severe AP may be as high as 25 percent [2]. In adults, the most common etiologies are biliary and ethanol-induced AP, whereas drug-induced AP (DIAP) is regarded as a rare and mild entity accounting for ~ 2–5% of AP episodes [3]. In contrast, DIAP is the leading risk factor for the first attack of AP in children [4]. Although DIAP is generally considered a mild disease, a recent study highlighted that patients with DIAP have a more severe disease course compared with AP of other etiologies [5], which may be explained by the preexisting pathologies and primary disease of DIAP patients. Even more importantly, the treatment of the primary disease usually has to be suspended or delayed due to DIAP, which can lead to relapses or an accelerated disease progression, thus prevention of DIAP is a major unmet need. A better understanding of the pathogenesis of DIAP, which presumably differs among different drugs, may help to overcome this severe side effect. As an example, Peng et al. recently described the pathomechanism responsible for asparaginase-induced AP, which opened preventive opportunities [6]. Asparaginase is an essential treatment for acute lymphoblastic leukemia – the most common malignant disease in children – with AP as a common adverse event occurring in about 5–10% of cases, which is the most frequent cause of discontinuing the asparaginase treatment [7]. Peng et al. demonstrated that similarly to other pancreatitis-inducing agents, the effect of asparaginase involves protease-activated receptor 2, evokes intracellular Ca^2+^ release followed by Ca^2+^ entry, and a reduction of Ca^2+^ extrusion due to decreased intracellular adenosine triphosphate levels, ultimately leading to extensive acinar cell necrosis [6]. In a follow-up manuscript, the authors also proved that galactose administration protects pancreatic acinar cells ex vivo and reduces the severity of AP in vivo, suggesting that galactose would be a valuable addition to the current asparaginase treatment protocol [8].

Another common form of DIAP is thiopurine-induced pancreatitis (TIP), which is one of the most frequent adverse drug reactions observed in patients with inflammatory bowel diseases (IBD) receiving azathioprine (AZA). TIP occurs in up to 8% of the cases [9–12], whereas AZA treatment increases the risk of AP fivefold compared to no treatment [13, 14]. Moreover, TIP requires the cessation of thiopurine therapy and the change of medication, often to a less effective drug [15], and might be associated with a higher risk of surgery [16]. After their development in the 1950s, the three thiopurines – AZA, 6-mercaptopurine (6-MP), and 6-thioguanine (6-TG) – became the cornerstones of treatment in organ transplantation, and a wide range of inflammatory, autoimmune, and hematological diseases, but the most experience has been gathered in the field of gastroenterology and the management of IBD [17, 18]. Thiopurines express their immunosuppressant effects through their active metabolites, 6-thioguanine-nucleotides, which can incorporate into the DNA and RNA [10], and inhibit the small GTPase, Ras-related C3 botulinum toxin substrate 1 (RAC1), triggering apoptosis [19, 20]. This effect is more pronounced in the rapidly dividing lymphocytes, hence the immunosuppressive effect; however, thiopurines have a quite narrow therapeutic window and their administration induces various adverse events up to a cumulative incidence of 26% [11]. As the pathomechanism leading to the development of TIP is currently not understood, preventive strategies cannot be developed.

Therefore in the current study, our aim was to understand the pathomechanism leading to the development of TIP. Here, we successfully recapitulated TIP in mice and demonstrated that AZA-treated animals are more susceptible to developing AP upon cerulein treatment. When assessing the possible mechanism of TIP, we found that AZA disturbs pancreatic ductal ion and fluid secretion in mouse pancreatic ductal epithelial cells (PDEC), but has no effect on pancreatic acinar cells. Notably, impaired pancreatic ductal secretion was shown previously to increase the severity of AP [21, 22], and we demonstrated that AZA inhibits the ductal Cl^–^, HCO_3_^–^, and fluid secretion in mouse pancreatic ductal cells both in the in vivo and ex vivo models. Importantly, this inhibition was due to an AZA-mediated suppression of RAC1 activity in mouse pancreatic cells leading to decreased co-localization of cystic fibrosis transmembrane conductance regulator (CFTR) and the anchor protein ezrin and impaired plasma membrane localization of CFTR. To our knowledge, this is the first study providing a detailed mechanistic understanding of TIP. Moreover, we argue that stimulation of pancreatic ductal secretion may help to reduce the risk of TIP in IBD patients.

## Materials and methods

All used materials with catalog numbers can be found in Tables 1 and 2.

**Table 1:** A list of used chemicals and materials

Name	Provider	Cat. No.:
Azathioprine	ThermoFisher Scientific	J62314
6-thioguanine	ThermoFisher Scientific	B21280
6-Mercaptopurine monohydrate	ThermoFisher Scientific	A12197
Stainless steel feeding tubes, 22ga × 25 mm, straight	Instech Laboratories	FTSS-22S-25
Human secretin	Sigma-Aldrich	S7147
Harrys Hematoxylin	Leica	3801560E
Eosin Y alcoholic solution	Leica	3801600E
DMEM/F-12 Ham	Sigma-Aldrich	D6421
Collagenase	Worthington	LS005273
Soybean trypsin inhibitor	Gibco	17,075,029
Bovine serum albumin (BSA)	Sigma-Aldrich	A8022
CELLSTAR 48-well culture plates, clear	Greiner Bio-One	M8937-100EA
x-well cell culture chamber, 8-well, on Glass slide	Sarstedt	94.6170.802
Cover glass	VWR	ECN 631–1583
Poly-L-lysine	Sigma-Aldrich	P4707-50ML
BCECF, AM	Invitrogen	B1170
MQAE	Invitrogen	E3101
FURA-2, AM	Invitrogen	F1201
Pluronic F-127	Invitrogen	P3000MP
Shandon cryomatrix	Thermo Scientific	6,769,006
Triton-X-100	Reanal labor	32,190–1-99-33
Sodium citrate	Sigma Aldrich	C8532
PFA	Alfa Aesar	43,368
PBS	Sigma-Aldrich	P4417-100TAB
Goat serum	Sigma-Aldrich	G9023
ProLong™ gold antifade mountant	Invitrogen	P36930
Anti-CFTR antibody, polyclonal, rabbit	Alomone Labs	ACL-006
Goat anti-rabbit IgG (H + L) highly cross-adsorbed secondary antibody, Alexa Fluor 488	Invitrogen	A-11034
Recombinant anti-ezrin antibody [EPR23353-55], monoclonal, rabbit	Abcam	ab270442
Anti-CFTR antibody [CF3]	Abcam	ab2784
Donkey anti-mouse IgG (H + L) highly cross-adsorbed secondary antibody, Alexa Fluor 647	Invitrogen	A-31571
Goat anti-rabbit IgG (H + L) cross-adsorbed secondary antibody, Alexa Fluor 568	Invitrogen	A-11011
Collagenase IV	Worthington	LS004188
Dispase	Sigma-Aldrich	D4693
Corning matrigel	Corning	354,234
Glucose oxidase	Sigma-Aldrich	G2133-50KU
6-well plates	Greiner BioOne	657,160
Catalase	Sigma-Aldrich	C100
Glucose	Sigma-Aldrich	G8270
Cysteamine hydrochloride	Sigma-Aldrich	M6500
Ehop-016	Sigma-Aldrich	SML0526
NSC668394	Sigma-Aldrich	341,216
RAC1 G-LISA activation assay	Cytoskeleton	BK128
alpha-amylase assay	Diagnosticum	47,462
Cerulein	Tocris Bioscience	6264
Apoptosis-necrosis detection kit (blue, green, red)	Abcam	ab176749
Eppendorf safe-lock tubes	Eppendorf	30,121,589
Subcellular protein fractionation kit for cultured cells	Thermo Scientific™	78,840
CellTiter-Glo® 3D cell viability assay	Promega	G9682

**Table 2:** Composition of solutions used during experiments

	Standard HEPES	Standard HCO_3_^–^	20 mM NH_4_Cl-HCO_3_^–^	Cl^–^-free HCO_3_^–^	Cat. No.: *(Sigma-Aldrich)*
NaCl	130	115	95		S9625
KCl	5	5	5		P4504
MgCl_2_ • 6H_2_O	1	1	1		M0250
CaCl_2_ • 2H_2_O	1	1	1		C3881
Hepes	10				H3375
D-(+)-Glucose	10	10	10	10	G8270
NaHCO_3_		25	25	25	S8875
NH_4_Cl			20		A4514
Na-gluconate				115	S2054
K_2_-sulfate				2.5	P0772
Ca-D-gluconate • H_2_O				6	G4625
Mg-D-gluconate • xH_2_O				1	G9130

HCO_3_^−^ containing solutions were gassed with 2 L/min carbogen. Concentrations are shown in millimolar (mM)

### Ethics

All experiments were performed in compliance with the Hungarian regulations and EU directive 2010/63/EU for the protection of animals used for scientific purposes and the study was approved by the National Scientific Ethical Committee on Animal Experimentation (approval nr: CS/I01/2233-4/2018).

### Animals

The C57BL6 mice used in this study were 8–12 weeks old and weighed 20–25 g. The gender ratio was 1:1 for all groups. Animals were allowed to have free access to chow and water, kept at a constant 24 °C room temperature and humidity, and under a 12-h light–dark cycle. Animals received VRF1(P) standard rodent food (Special Diets Services, UK, Cat.No.: 801900), and standard bedding (JRS, Germany; REHOFIX MK2000 corn cob) purchased from Akronom, Hungary.

### In vivo experimental pancreatitis

Moderate experimental pancreatitis was induced with 8 × 50 μg/body weight in kilograms (kgbw) IP cerulein (Tocris Bioscience, UK) injections administered hourly, as described previously [22, 23]. Sham control animals received intraperitoneal (IP) physiologic saline injections. Mice were sacrificed one or four hours after the last injection unless otherwise stated, then pancreata were removed, washed with physiologic saline, cleaned from lymph nodes and fat, and stored at + 4 °C in 4% formaldehyde. Paraffin-embedded sections of the pancreas were stained with hematoxylin–eosin following standard protocol. During the histologic evaluation, the severity of pancreatitis was assessed independently by three observers, blinded for the experimental setup, based on the histologic scores of 3 random fields per slide. The extent of the tissue edema, and leukocyte infiltration, were scored from 0 to 5 and the proportion of acinar cell necrosis was evaluated as previously described [21]. Images were captured with a Zeiss AxioImager.M2 with 5× objective (N-Achroplan 5x/0.15 M27; Zeiss, Germany). To determine the serum amylase activity, blood samples were collected into tubes containing no anticoagulants by cardiac puncture under ketamine-xylazine anesthesia. Within an hour after collection, blood samples were centrifuged at 2000 × g for 20 min at 4ºC, then stored at -20 °C until activity measurements or used immediately. To determine the serum alpha-amylase activity, a 4,6-ethylidene(G1)-4-nitrophenyl(G7)-a-(1–4)-D-maltoheptaoside (EPS)-based kinetic assay was used following the manufacturer’s protocol (Diagnosticum Zrt, Hungary, see Table 1.). Briefly, 20 µL serum sample was diluted and from the diluted sample 4 µL was pipetted onto a 96-well plate, then samples were incubated at 36 °C with the alpha-glucosidase containing reagent for 3 min and the reaction was started with the application of the EPS substrate solution; the sample-reagent ratio was 1:75, the enzyme–substrate ratio was 5:1. After 3 min shaking of the plates, the absorbance (A) of the wells was measured at 410/505 nm for 20 min with a CLARIOStar plate reader (BMG Labtech, Germany), and the slope of absorbance (∆A/min) was determined for each well using blank corrected values in the MARS Software (BMG Labtech, Germany). Amylase activity was calculated according to the equation given by the assay manufacturer (7280 × ∆A/min = 1 IU/ml) and standardized to body weight and serum protein content. Before the measurements, the optimal dilution of samples was determined to ensure that the amylase activity was within the linear range of the assay.

### *Per os* treatment of mice with thiopurines

Based on previous pharmacokinetic studies, the usual therapeutic serum concentrations of AZA were below 1 μg/mL in human subjects treated with conventional oral AZA doses (0.5–2.5 mg/kgbw/day) [24], and the maximal serum levels in mice were observed to be 11.3 μg/mL after a single oral dose of 33.3 mg/kgbw AZA [25]. To test both therapeutic and toxic concentrations of thiopurines, we chose to use the 1 and 10 μg/mL concentrations in most of the ex vivo experiments, while to assess the in vivo thiopurine exposure, 1.5 and 15 mg/kgbw in vivo doses were used. Briefly, mice received a daily dose (150 μL) of either thiopurine (1.5 or 15 mg/kgbw/day AZA, 6-MP, or 6-TG) or sterile physiologic saline as sham control, for one or four weeks through a 22 G gastric feeding tube (Instech Laboratories, PA, USA), respectively. After the oral treatment, mice were selected either for further experiments or immediate pancreas collection and cell isolation.

### Measurement of in vivo pancreatic fluid secretion

The stimulated in vivo pancreatic juice-secretion was measured in ketamine-xylazine sedation, as previously described [26]. Briefly, through a narrow laparotomy, the duodenum and the head of the pancreas were exposed and the main pancreatic duct was cannulated with a blunted 26 G needle and fixated with a vascular clip. To avoid measuring the bile secretion, the ductus choledochus was also clipped carefully. Total pancreatic juice secretion was stimulated using IP 0,75 U/kgbw human secretin and the secreted volume was measured after 30 min with a 100 μL pipette and normalized to the animals' bodyweight and time.

### Isolation of pancreatic acinar cells and ductal fragments

Mouse pancreatic acinar cell clusters were isolated as previously described [27, 28], with some adaptations. Briefly, the collected pancreas was minced into 1–3 mm^3^ pieces and placed in ice-cold HBSS. Lipids and fats were removed by centrifugation at 450 × *g* for 2 min. Tissue pieces were digested in 5 mL freshly prepared HBSS solution containing 200 units/ml collagenase, 10 mM HEPES and 0.25 mg/ml trypsin inhibitor at 37 °C for 20–30 min. Digested tissue was washed with HBSS of 4 °C with 10 mM HEPES, 0.25 mg/ml soybean trypsin inhibitor, and 5% FBS, centrifuged 3 × for 2 min at 450 RCF (Rotor radius: 180 mm), and the pellet was filtered through a 100 μM cell mesh and resuspended in Medium-199 with 2.5% FBS and 0.25 mg/ml soybean trypsin inhibitor. Pancreatic ductal fragments were isolated as described previously [29, 30]. Briefly, mice were euthanized with pentobarbital, and the pancreas was removed, and placed into ice-cold DMEM/F12. The pancreas was injected with a digestion medium (DMEM/F12 containing 100 U/ml collagenase, 0.1 mg/ml trypsin inhibitor, and 1 mg/ml bovine serum albumin) and was incubated in a shaking water bath at 37ºC for 30 min. Small intra- and interlobular ducts were identified and were mechanically dissected from the acini under a stereomicroscope.

### Preparation mouse pancreatic organoid culture

Mouse pancreatic ductal organoids were generated as previously described [22, 29]. Briefly, the mouse pancreas was minced into small fragments and incubated for 1 h at 37 °C in a digestion media containing collagenase IV and dispase. After digestion, the tissue was washed and resuspended in Matrigel Basement Membrane Matrix and plated in domes and kept in culture (at 37 °C 95% relative humidity, and 5% CO_2_) by changing media every other day. Organoids were used for experiments between passage number 2 and 5. For the experiments, organoids were digested into single cells TrypLE Express and after 60 min of recovery time, they were incubated in 6-well plates with drug/inhibitor-containing or control culture media for another 60 min.

### Measurement of intracellular pH, Ca^2+^, and Cl^–^ changes by fluorescence microscopy

Fluorescence microscopy measurements were taken as previously described [21–23]. Isolated pancreatic acini or ductal fragments were attached to a poly-L-lysine coated cover glass, placed in a perfusion chamber, and incubated with MQAE (2 μM), Fura 2-AM (5 μM), or BCECF-AM (2 μM) in HEPES solution for 30 min at 37 °C with 5% CO_2_. After incubation, chambers were mounted on an Olympus IX73 inverted microscope (Olympus, Japan) and were perfused continuously at a constant speed with extracellular solutions heated to 37 °C. Microscopes were equipped with LED illumination systems (Olympus CoolLED PE-4000 and pE-340^Fura2^) and matching dichroic filters. A Hamamatsu Orca-Flash 4.0 CCD camera (Hamamtsu Photonics, Japan) and an Olympus 20× water immersion objective (NA: 0.8) were used for capturing fluorescent signals with a temporal resolution of 1 s using the Olympus Excellence software. To test the effect of thiopurines and inhibitors we changed the perfusion from control solutions to thiopurine and/or inhibitor-containing buffered solutions for 10 min before and during performing the stimulations described below. The first n number defines the number of animals, the second is the number of independent experiments and the third is the number of acinar cells/ductal fragments analyzed.

Intracellular Ca^2+^ signals ([Ca^2+^]_i_) were measured as described previously [22, 29], during the continuous recording of the fluorescent signal captured from acinar cells, as well as from PDEC loaded with FURA-2 dye. One region of interest (ROI) was placed on one acinar cell, therefore the number of ROIs is the same as the acinar cell numbers. The cells were continuously perfused either with control or 100 nM Carbachol containing standard HEPES solutions (Table 2). The fluorescence signals were normalized and expressed as relative fluorescence (F/F_0_).

To estimate the Cl^**–**^ secretory activity of PDEC, we continuously monitored the intracellular Cl^**–**^ concentration ([Cl^**–**^]_i_) of the ductal fragments with the help of the Cl^**–**^-sensitive fluorescent dye MQAE, during continuous perfusion with HCO_3_^**–**^/CO_2_^**–**^-buffered solutions. The loaded cells were excited with 340 nm. The increase in the fluorescent intensity reflects a decrease in the intracellular Cl^−^ concentration. Initially, ductal fragments were perfused with standard HEPES solutions for a brief period to wash out the excess fluorescent dye, then the perfusion was changed to standard HCO_3_^**–**^-solution (Table 2). After reaching a steady-state fluorescent signal, the extracellular Cl^–^ was removed by switching to Cl^–^-free HCO_3_^−^-solution (Table 2). The removal of extracellular Cl^**–**^ induces a rapid elevation in fluorescence intensity equivalent to a decrease in [Cl^**–**^]_i_, and the relative amplitude of this change is proportionate to the luminal Cl^**–**^ efflux mediated by CFTR. The fluorescence signals were normalized and expressed as relative fluorescence (F/F_0_).

*Recovery from an alkaline load.* Initially, ductal fragments were perfused with standard HEPES solutions for a brief period to wash out the excess fluorescent dye, then the perfusion was changed to standard HCO_3_^–^-solution (Table 2). Then, during continuous perfusion with HCO_3_^–^/CO_2_-buffered solutions, we monitored the changes in intracellular pH (pH_i_). During alkaline loading, ductal fragments were exposed to 20 mM NH_4_Cl from the basolateral side, where the passive influx of NH_3_ triggered a rapid alkalosis of the pH_i_, followed by a decaying recovery (downward-tilted arrows in Figs. 3A, 4A, D) created by the active efflux of HCO_3_^–^ through luminal SLC26A and CFTR proteins. The initial rates of recovery (dpH_i_/dt) over 30 s from the highest pH_i_ levels were calculated and converted to base flux values (B^–^/min) using calibration data from previously published measurements [21, 29, 31].

*Recovery from an acid load.* Ductal fragments were loaded with NH_4_Cl as described above. The removal of NH_4_Cl from extracellular solution resulted in an unfacilitated NH_3_ efflux, which triggered rapid acidification in the pH_i_, followed by a decaying increase in the pH_i_ (upward-tilted arrows in Figs. 3A, 4A, D) created by the active influx of HCO_3_^–^ and the active efflux of H^+^ through the basolateral NBCe1 and NHE1&4 proteins, respectively. The initial rates of recovery (dpH_i_/dt) over 60 s from the lowest pH_i_ levels were calculated and converted to base flux values (B^–^/min) using calibration data from previously published measurements [21, 29, 31].

### Immunostaining

Immunofluorescent labeling on sectioned ductal fragments was performed as previously described [29]. Briefly, isolated pancreatic ductal fragments and organoids were frozen in Shandon Cryomatrix at -20 °C. The 7-μm thick sections were placed on microscope slides, and fixation was performed with 4% PFA-PBS for 20 min followed by washing for 3 × 5 min with PBS. After permeabilization in citrate/Triton-X 100, the sections were blocked with 0.1% goat serum and 10% BSA in PBS for 2 h. The overnight incubation with primary CFTR (rabbit, monoclonal) antibodies at 4 °C was followed by repeated washing steps after which secondary (goat anti-rabbit Alexa Fluor 488) antibodies were added for 2 h at room temperature. Nuclear staining and mounting were carried out simultaneously by ProLong™ Gold Antifade mounting medium with DAPI. Images were captured with a Zeiss LSM880 confocal microscope using a 40× oil immersion objective (Plan-Apochromat 40×/1.4 Oil DIC M27; Zeiss, Germany). For the semiquantitative analysis, images were captured with the **s**ame setup from 5 different ductal fragments per group, derived from at least 2 different mice. On each image, CFTR fluorescence intensity profiles were determined over 4.5 μm long vectors at every 30 μm all around the identified luminal cavities, starting from the luminal side. Linear profiles were determined using the FIJI software (NIH, USA), peak intensity was noted in arbitrary units (AU), and peak distance from the lumen, as well as peak width (measured at half-maximal intensity), was calculated. The peak distance value was calculated starting from the first point with an intensity of less than 0.3 AU.

### Direct stochastic optical reconstruction microscopy (dSTORM)

Primary mouse pancreatic ductal cells were generated for dSTORM by digesting and plating mouse pancreatic ductal organoids on cover glass followed by incubation for 60 min with 1 μg/mL AZA-containing media. Organoids were fixed with 4% PFA in PBS for 10 min and antigen retrieval was performed with 0.01% Triton-X-100 in PBS for 10 min. Specific binding sites were blocked by applying 10% BSA in PBS for 2 h at 37 °C. CFTR and ezrin primary antibodies (1:100) were applied during overnight incubation at 4 °C and fluorophore-conjugated secondary antibodies were applied (mouse anti-rabbit Alexa Fluor 647 and goat anti-rabbit Alexa Fluor 568). Cover glasses were placed in blinking buffer (which contains 100 U glucose oxidase, 2000 U catalase, 55.5 mM glucose, and 100 mM cysteamine hydrochloride in 1 mL final volume with sterile PBS) and dSTORM images were captured by Nanoimager S (Oxford Nanoimaging ONI Ltd., UK). Cluster analysis of dSTORM images was evaluated by CODI (Oxford Nanoimaging ONI Ltd., UK). A channel-independent cluster analysis was performed based on the collected and individually recorded blinking events. Clusters were defined using a diameter of 120 nm from the center. The number of homogeneous and double-positive clusters was determined by examining the composition of individual clusters.

### Biochemical assays

*RAC1 Activity.* Mouse pancreatic organoids were digested into single cells using TrypLE™ Express and incubated at 37 °C with 1 μg/mL AZA, 10 μM Ehop-016, or both for 60 min. Cell lysates were harvested and RAC1 activity was determined with a colorimetric RAC1 G-LISA kit (Cytoskeleton, USA) following the manufacturer’s protocol. Briefly, the assay utilizes Rac-GTP-binding protein immobilized to the bottom of a 96-well plate, which specifically binds to the active GTP-bound Rac1, while the inactive GDP-bound Rac1 is not interacting with the binding protein and is removed during washing. Then the active Rac1 is quantified with a Rac1-specific antibody. The degree of Rac1 activation is calculated from the ratio of the activated cell lysates and inactive cell lysates.

*Amylase release assay.* Freshly isolated acinar cells were seeded to 48-well plates and incubated for 30 min at 37 °C with supplemented Medium-199, then 1 μg/mL AZA and/or 100 nM cerulein-containing media was added, and cells were incubated for another 60 min. After incubation, aliquots were taken from the supernatant and frozen instantly in liquid nitrogen to calculate the released amylase activity. Then, Triton X-100 was added in a final concentration of 1% to the remaining cell suspension, incubated for 15 min, centrifuged at 450 × g for 3 min, 4 °C, and a second aliquot was frozen in liquid nitrogen to calculate the total amylase activity. Both activities of released and total amylase were determined for each sample using an EPS method-based[32] Alpha-Amylase Kit (Diagnosticum Zrt., Hungary) on a CLARIOStar plate reader at 405 nm (BMG Labtech, Germany) following the manufacturer's protocol, and the percentage of released amylase was calculated.

### Measurement of cell viability

Freshly isolated acinar cells were incubated for 30 min with supplemented Medium-199 in 48-well plates (Greiner Bio-One, Hungary), then AZA containing (0.1–1-10–100-1000 μg/mL) media was added, and cells were incubated for another 60 min at 37 °C. Cerulein (100–1000 nM) was applied as positive control. We determined the intracellular ATP content, which is proportional to the number of viable cells, using the CellTitre-Glo 3D (Promega) luminometric assay on a CLARIOStar plate reader. The total protein amount was determined using by Bradford assay in Spectrophotometer (Thermo Scientific™ NanoDrop™ One). Blank-corrected data were normalized to the total protein amount.

In addition, living, apoptotic, and necrotic cells were stained in 8-well chamber slides (Sarstedt, Germany) with Apoptosis-Necrosis Kit (Abcam) and were visualized under a Zeiss LSM880 confocal microscope using a 40× oil immersion objective (Zeiss, NA: 1.4). The total number of cells was calculated in FIJI (NIH, USA) using the built-in Cell Counter plugin and the ratio of the live, apoptotic and necrotic cells were given as the % of the total cell number.

### Statistical analysis, post-tests

All experimental data were expressed as means ± SD. One-way ANOVA followed by Sidak’s post hoc test was used for multiple group comparisons. The chi-square test was used for the comparison of frequencies. T-test was used for pairwise comparisons. Two-way ANOVA with Tukey’s post hoc test was used for comparisons of multiple groups split by independent variables. *P* < 0.05 was accepted as being significant. All authors had access to the study data, and reviewed and approved the final manuscript.

## Results

### AZA treatment increases pancreatic damage in the early phase of cerulein-induced pancreatitis in mice

**Fig. 1:**
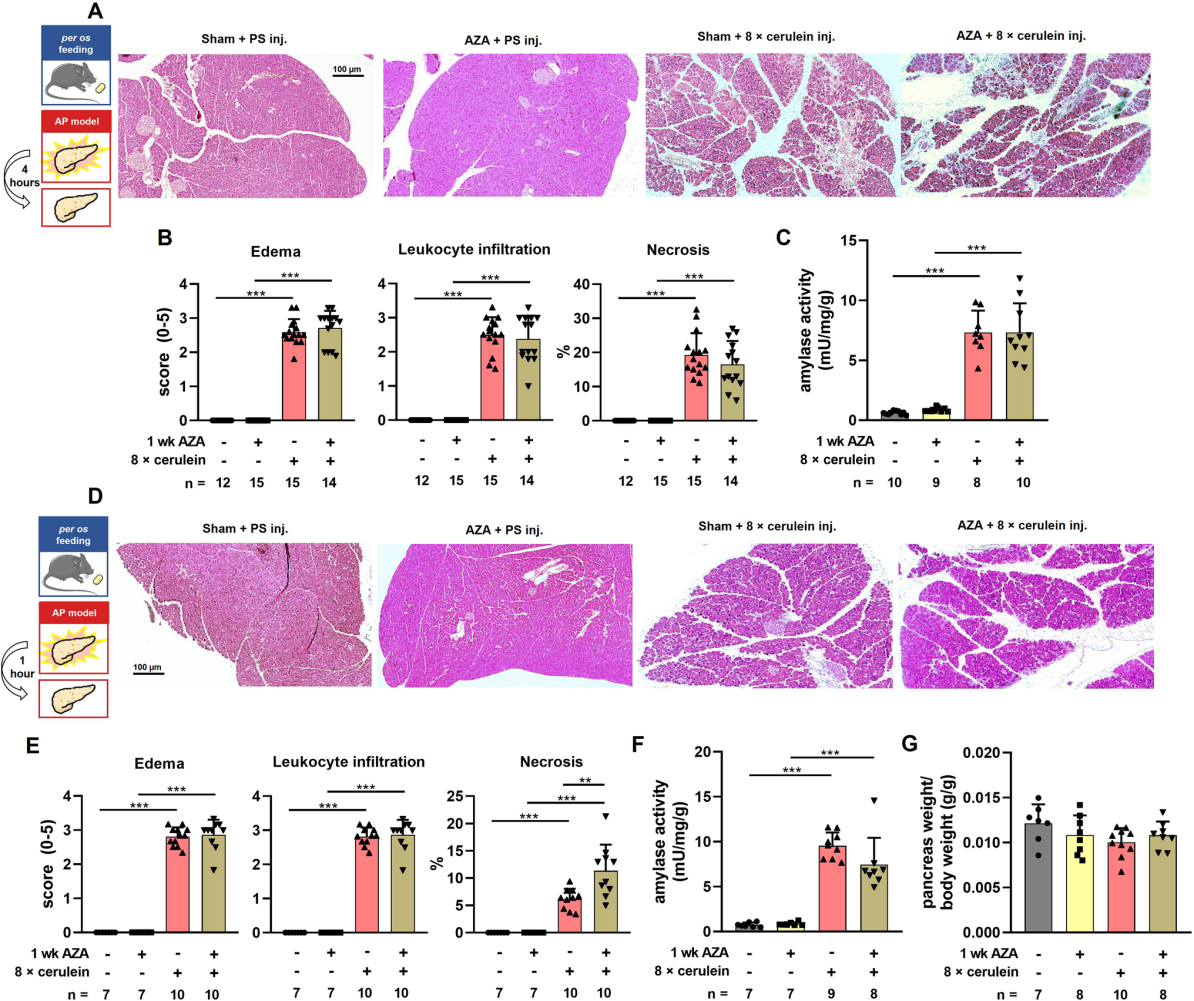
AZA treatment increases the early pancreatic damage in cerulein-induced acute pancreatitis in mice. Mice received either 1.5 mg/kgbw daily oral doses of azathioprine (AZA) or *per os* physiologic saline (Sham) for one week, followed by induction of moderate experimental pancreatitis through the administration of 8 × cerulein IP injections. Controls received 8 × IP physiologic saline (PS) injections. Animals were sacrificed 4 (**A**-**C**), or 1 (**D**-**G**) hour after the last cerulein injection. **A** Representative hematoxylin–eosin (**H**-**E**) stained slides of formaldehyde-fixed pancreata captured with 50 × total magnifications and corrected for background illumination. **B** Histology scores and percentage of necrosis of H-E stained slides in moderate experimental pancreatitis (*n* = *15, sham* + *PS; n* = *15, AZA* + *PS; n* = *15, sham* + *cerulein; n* = *14, AZA* + *cerulein groups*). Each slide was analyzed in at least 3 different fields of view, by three independent observers. **C** Activity of serum amylase in moderate experimental pancreatitis (*n* = *10, sham* + *PS; n* = *9, AZA* + *PS; n* = *8, sham* + *cerulein; n* = *10, AZA* + *cerulein groups.*). The data are shown as mean ± SD*,* *P < 0.05, ***P < 0.001, one-way ANOVA with Sidak’s multiple comparisons tests. **D** Representative hematoxylin–eosin (H-E) stained slides of formaldehyde-fixed pancreata captured with 50 × (large pictures) and 100 × (inserts) total magnifications and corrected for background illumination. **E** Histology scores and percentage of necrosis of H-E stained slides in moderate experimental pancreatitis (*n* = *7, sham* + *PS; n* = *7, AZA* + *PS; n* = *10, sham* + *cerulein; n* = *10, AZA* + *cerulein groups*). Each slide was analyzed in at least 3 different fields of view, by three independent observers. **F** Activity of serum amylase in moderate experimental pancreatitis (*n* = *7, sham* + *PS; n* = *7, AZA* + *PS; n* = *9, sham* + *cerulein; n* = *8, AZA* + *cerulein groups.*). **G,** The bar graph shows the pancreas weight/body weight ratio. (*n* = *7, sham* + *PS; n* = *8, AZA* + *PS; n* = *10, sham* + *cerulein; n* = *8, AZA* + *cerulein groups.*) Scale bar: 100 µm. The data are shown as mean ± SD*,* **P < 0.01, ***P < 0.001, one-way ANOVA with Sidak’s multiple comparisons tests

First, we wanted to model TIP in an in vivo animal model. To achieve this, mice were treated *per os* with 1.5 mg/kgbw/day AZA (a conventional human daily dose, [33]) for 1 week, then moderate AP was induced by 8 × 50 μg/kgbw IP cerulein injections. When the mice were sacrificed 4 h after the last cerulein injection, we were not able to observe any significant changes in the severity of AP in the AZA pre-treated group, as neither the average histological scores (Fig. 1A, B) nor the average serum amylase activities (Fig. 1C) of the cerulein-treated groups were significantly different. Also, when the mice were terminated 1 h after the last cerulein injection, the extent of interstitial edema, leukocyte infiltration, average pancreas weight/body weight ratio, or serum amylase activity were not significantly different in the cerulein-treated groups (Fig. 1D-G). However, the extent of necrosis was significantly higher in the AZA + cerulein-treated group compared to cerulein-treated groups (Fig. 1E). To test the effects of AZA on the severity of AP in an independent model, AP was also induced by 2 hourly injections of 1.35 g/kgbw ethanol + 150 mg/kgbw palmitoleic acid (POA) after 1 week *per os* 1.5 mg/kgbw/day AZA treatment. The animals were sacrificed 24 h after the first ethanol + fatty acid injection. Similarly to the results from the cerulein-induced model, there are no significant differences were observed in histological parameters (Suppl. Figure 1A-B), serum amylase level (Suppl. Figure 1C), and pancreas weight/body weight ratio (Suppl. Figure 1D) between ethanol + AZA-treated and alone ethanol-treated mice. These results suggest that we successfully modeled the effect of AZA treatment on the exocrine pancreas, which can increase tissue damage in the early phase of AP the disease.

### AZA has no detectable effect on pancreatic acinar cells in mice

**Fig. 2:**
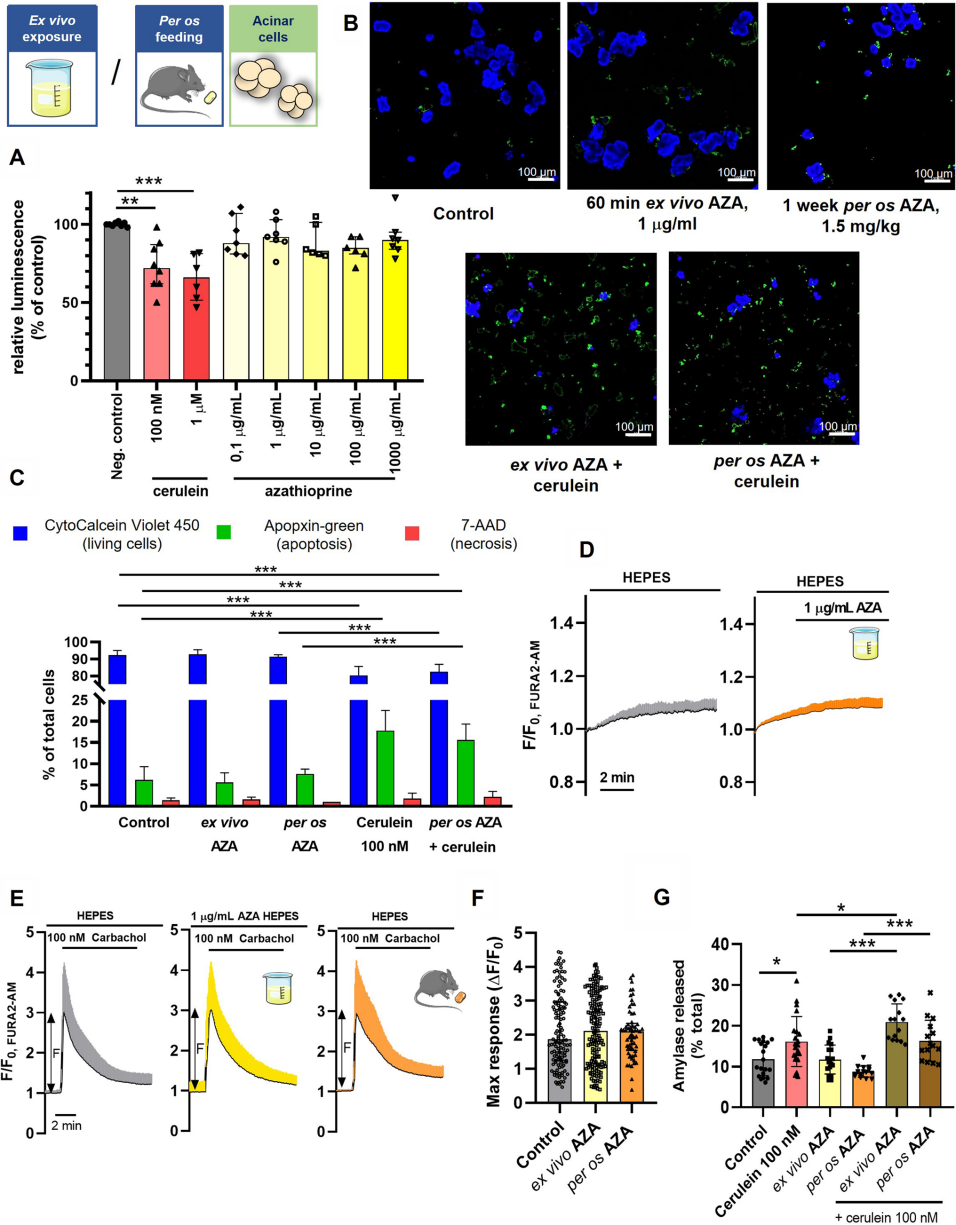
AZA has no detectable effect on pancreatic acinar cells in mice. **A** The proportion of viable mouse pancreatic acinar cells was measured after incubation with different concentrations of either AZA or cerulein for 60 min in 48 well plates in vitro. The total protein amount was determined using by Bradford assay in Spectrophotometer (Thermo Scientific™ NanoDrop™ One). Blank-corrected data were normalized to the total protein amount. The relative luminescence of wells was measured with CellTitre Glo 3D luminometric assay. Recorded luminescence of wells relative to control wells is shown as mean ± SD, *n* = 6–8 wells, measured in triplicates, from at least 3 animals for each group; **P < 0.01, ***P < 0.001, one-way ANOVA with Sidak’s multiple comparisons tests. **B**, **C** Cell death and viability were assessed on acinar cells from *per os* azathioprine (AZA) treated or control animals, with or without 60 min of incubation with 100 nM cerulein and/or 1 μg/mL AZA in 8-well chamber slides, using a fluorescent staining kit. **B** Representative fluorescent images of apoptosis-necrosis staining of acinar cells, captured with an LSM880 confocal microscope using a 40× oil immersion objective. **C** Average proportion of living, necrotic and apoptotic cells in the different treatment conditions, shown as a percentage of total cells (n = 5 fields of view, in total ca. 1500–2000 cells, from 2 animals per group). The data are shown as mean ± SD, ***P < 0.001, two-way ANOVA with Tukey’s multiple comparisons tests. **D-F** For Ca^2+^ measurements, mouse pancreatic acinar cells, either from untreated animals or from *per os* AZA-treated animals, were ex vivo stimulated with carbachol, with or without a previous 10-min long perfusion with 1 μg/mL AZA. The response to carbachol stimulation in the intracellular Ca^2+^ concentration was monitored by recording the normalized FURA2 fluorescence. **D** Average traces of normalized FURA2 fluorescence intensity during normal conditions and AZA exposure of pancreatic acinar cells. **E** Average traces of normalized FURA2 fluorescence intensity during carbachol stimulation of pancreatic acinar cells. The double-sided arrows mark how the maximal response values plotted on the F panel were calculated. **F** The magnitude of the maximal Ca^2+^ response to carbachol stimulation. The first n number defines the number of animals, the second number the number of independent experiments, and the third number the number of acinar cells analyzed (*n* = 3/7/125, control; *n* = 6/10/150, ex vivo AZA; *n* = 4/6/61). The data are shown as mean ± SD*, P* = 0.076, one-way ANOVA. **G**, Amylase release was measured after in vitro 60 min incubation of pancreatic cells in 48-well plates with cerulein or cerulein + 1 μg/mL AZA, and amylase activity of the supernatant and the total well after lysis of cells were measured with a colorimetric assay, in triplicates. The amylase release was measured also on acinar cells from *per os* AZA-treated mice (1 week, 1.5 mg/kg). The proportions of released amylase of acinar cells are plotted (*n* = 15–20 wells, from 3 animals in each group). The data are shown as mean ± SD*,* *P < 0.05, ***P < 0.001, one-way ANOVA with Sidak’s multiple comparisons tests

Premature activation of digestive enzymes due to pancreatic acinar cell injury and necrosis is a hallmark of AP with different etiologies, including forms of DIAP [34]. AZA is known to induce apoptosis in immune cells via a caspase-9-dependent pathway [35], therefore, we next investigated the effect of AZA on pancreatic acinar cell viability. For this, we incubated isolated pancreatic acinar cells with clinically relevant (0.1–1 μg/mL) and toxic (10–1000 μg/mL) concentrations of AZA for 60 min at 37ºC*.* None of the applied AZA concentrations were found to cause any significant change in the proportion of the living acinar cells, evaluated with a luminescence-based ATP assay (CellTitre^Glo^ 3D) compared to the positive control cerulein (Fig. 2A). Then, to compare acute and chronic AZA exposure we determined the proportion of viable, apoptotic, and necrotic/late apoptotic acinar cells after incubation with 1 μg/mL AZA for 1 h at 37ºC using an apoptosis/necrosis detection kit. In addition, we also isolated pancreatic acinar cells from in vivo AZA-treated mice using the same conditions as above (Fig. 2B, C). According to these experiments, AZA had no effect on the acinar cell viability either upon *per os* or ex vivo exposure. In contrast, the positive control cerulein markedly reduced the viability of the acinar cells as expected.

Most pancreatitis-inducing agents trigger acinar cell damage via sustained intracellular Ca^2+^ elevation [36, 37], whereas AZA was also found to increase the cytosolic Ca^2+^ levels ([Ca^2+^]_i_) in blood cells [38]. Therefore in the next step, we analyzed the effect of AZA treatment on the Ca^2+^ homeostasis of acinar cells. In these series of experiments, no effect of AZA was found on the Ca^2+^ signaling in acinar cells. The 10 min ex vivo perfusion of isolated pancreatic acinar cells with 1 μg/mL AZA was not sufficient to trigger Ca^2+^ signals (Fig. 2D), whereas the response of acinar cells to 100 nM carbachol in the presence of AZA was not altered (Fig. 2E, F). Similarly, we also could not observe any altered acinar Ca^2+^ signals after the 1-week-long in vivo AZA treatment**.**

Finally, to exclude the possible effect of AZA on pancreatic acinar cell functions, we determined the proportion of released amylase from the acinar cells. Cerulein was used as a positive control. In this series of experiments neither 60 min of incubation with 1 μg/mL AZA nor 1-week *per os* treatment with 1.5 mg/kgbw AZA was sufficient to induce amylase release (Fig. 2G). The co-stimulation of acinar cells with AZA and cerulein caused a moderate, but significant increase in amylase release compared to cerulein stimulation alone. On the other hand, the *per os* AZA-treated acinar cells did not recapitulate this effect. These results support that AZA has no pathophysiologically relevant effect on the pancreatic acinar cells.

### Thiopurines impair mouse pancreatic ductal HCO_3_^–^ secretion ex vivo

**Fig. 3:**
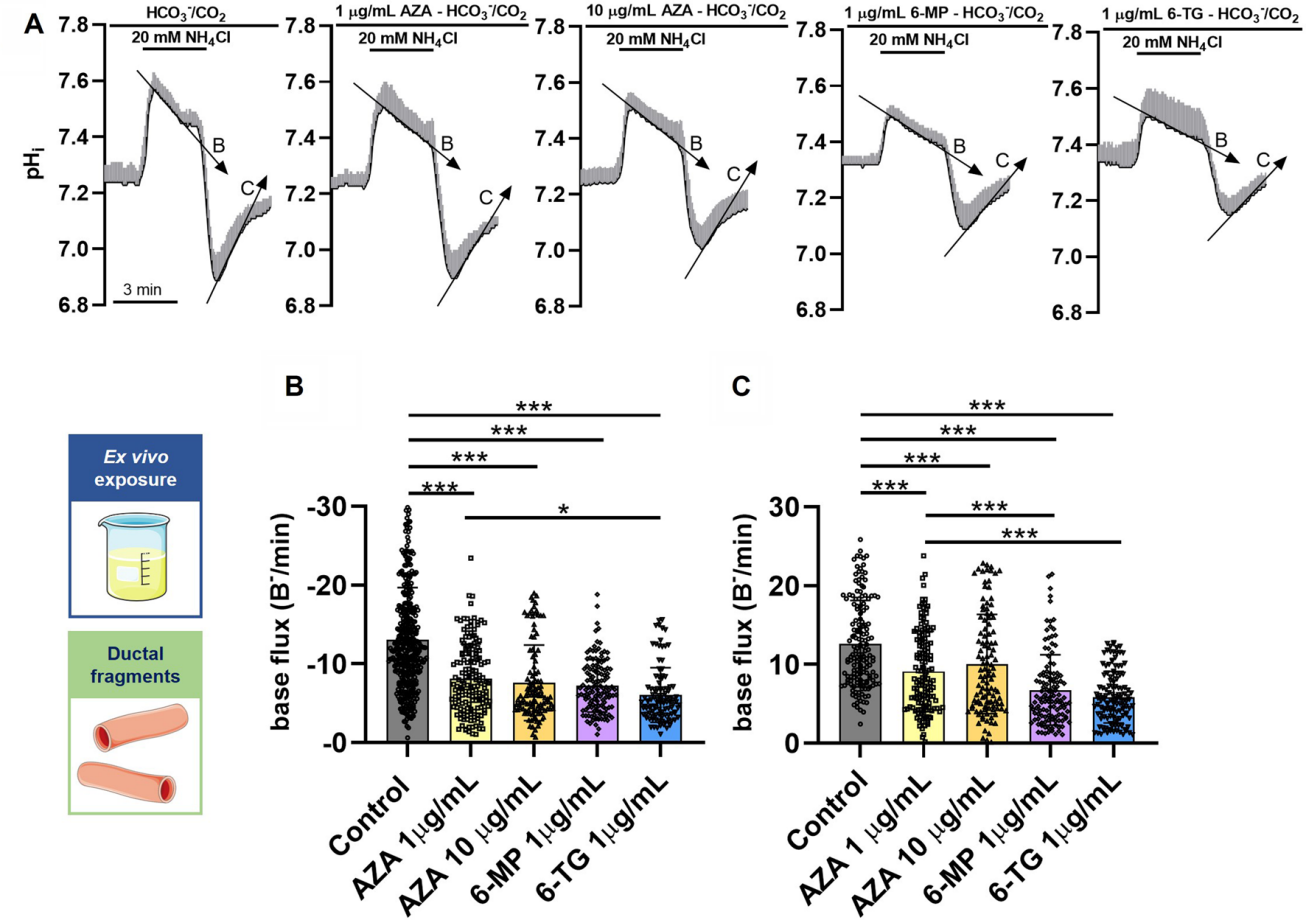
Ex vivo perfusion of mouse pancreatic ductal fragments with thiopurines inhibits HCO_3_^–^ secretion. Untreated mouse pancreatic ductal fragments were stimulated ex vivo with 20 mM NH_4_Cl (controls) then they were perfused for 10 min with 1 μg/mL or 10 μg/mL azathioprine (AZA), or 1 μg/mL 6-mercaptopurine (6-MP) or 6-thioguanine (6-TG), and the NH_4_Cl stimulation was repeated. BCECF-AM fluorescence signals were recorded and converted into intracellular pH (pH_i_) values. **A** The average traces (± SD) of intracellular pH, recorded during the NH_4_Cl stimulation. Downward- and upward-tilted arrows on the traces mark the slope of recoveries, which made the basis when calculating the base flux values displayed on B and C panels, respectively. **B**, **C** The base flux values during recovery from alkaline (**B**) and acid loading (**C**). The number of animals (*N*), ductal fragments (*n*), and regions of interest (ROIs) used in the experiments: 1 μg/mL AZA (*N* = 5, *n* = 15, ROIs = 157), 10 μg/mL AZA (*N* = 4, *n* = 12, ROIs = 111), 1 μg/mL 6-MP (*N* = 4, *n* = 12, ROIs = 129), or 1 μg/mL 6-TG (*N* = 4, *n* = 12, ROIs = 104). The data are shown as mean ± SD, *P < 0.05, ***P < 0.001, one-way ANOVA test with Sidak’s multiple comparisons tests

Pancreatic ductal cells secrete an alkaline fluid, which plays a major role in the maintenance of exocrine pancreatic homeostasis [22, 39]. As acinar cells were not affected by AZA, we wanted to assess the possible effect of AZA on the ductal cells. To estimate the HCO_3_^**–**^ transport in PDEC, we perfused isolated pancreatic ductal fragments with 1 and 10 μg/mL AZA for 10 min and found that the base flux values were significantly decreased during recovery from alkaline and acid load (Fig. 3A-C). The inhibitory effect was not dose-dependent in the investigated concentration range. Active metabolites of AZA (6-MP and 6-TG) were also reported to induce DIAP [40, 41], therefore, we tested the effect of all thiopurines on ductal HCO_3_^**–**^ secretion by perfusing the ductal fragments with 1 μg/mL 6-MP and 6-TG, respectively. Similar to AZA treatment, the base flux values during recovery from alkaline and acid load were significantly reduced by both 6-MP and 6-TG perfusions compared to controls (Fig. 3A-C). These results suggest that AZA and its active metabolites disturb the apical and basolateral HCO_3_^–^ transport processes in pancreatic ductal cells.

### Thiopurines impair pancreatic ductal HCO_3_^–^ secretion in vivo in mice

**Fig. 4:**
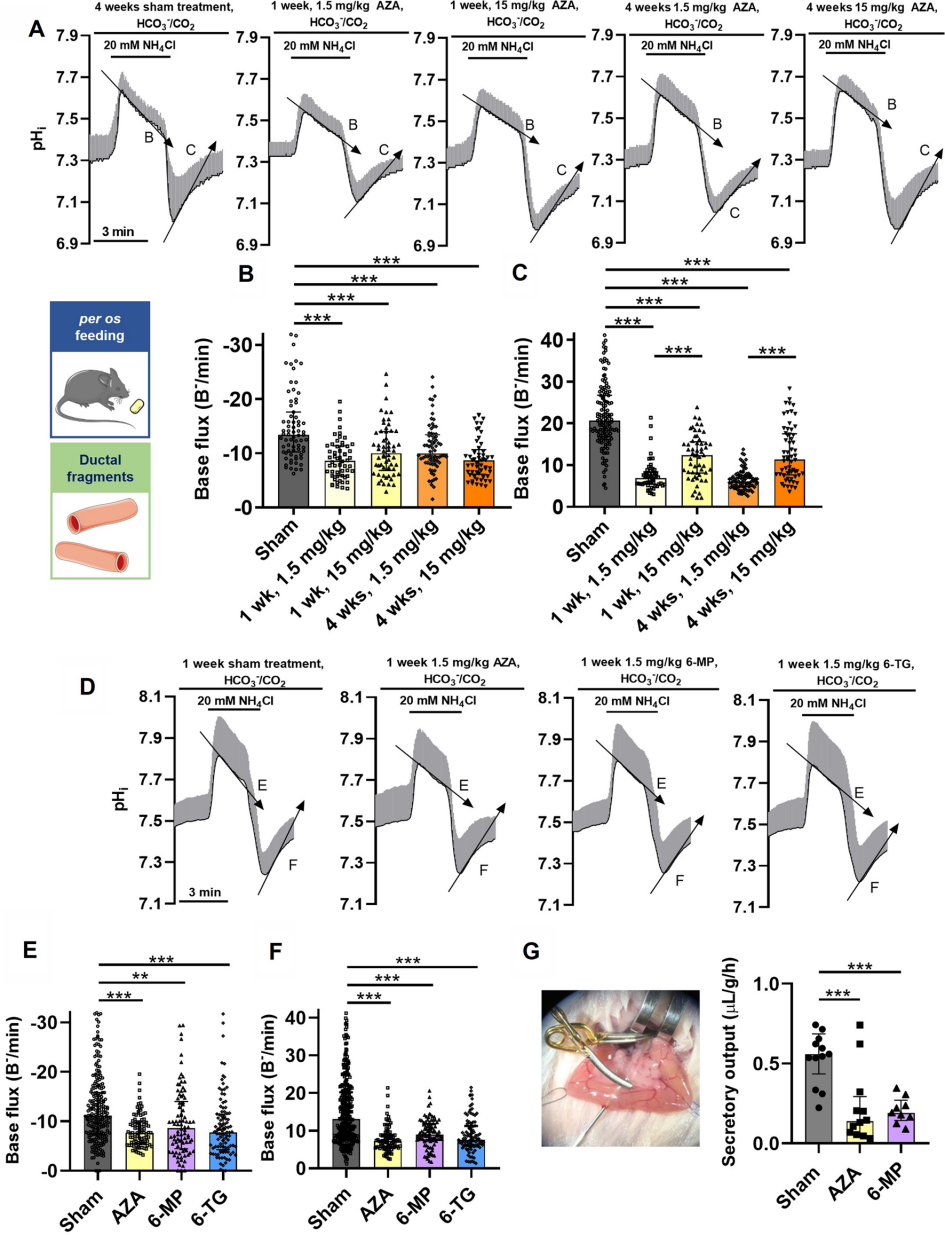
*Per os* treatment with thiopurines inhibit mouse pancreatic ductal HCO_3_^–^ secretion and pancreatic juice secretion. Mice received either 1.5 mg/kgbw daily oral doses of thiopurines, 15 mg/kgbw/day azathioprine (AZA), or 50 mL/kgbw/day physiologic saline (Sham). Isolated pancreatic ductal fragments were stimulated ex vivo with 20 mM NH_4_Cl while BCECF-AM fluorescence signals were recorded and converted into intracellular pH (pH_i_) values. The average traces (± SD) of pH, were recorded during the NH_4_Cl stimulation of ductal fragments from only AZA-treated (**A**) or thiopurine-treated (**D**) animals. **B**, **C** The base flux values during recovery from alkalosis (**B**, **E**) and acidosis (**C**, **F**), after treatment with different doses and durations of thiopurine, respectively. The number of animals (*N*), ductal fragments (*n*), and regions of interest (ROIs) used in the experiments **A-C** 4 weeks physiologic saline (*N* = 4, *n* = 12, ROIs = 71), 1 week 1.5 mg/kg AZA (*N* = 3, *n* = 10, ROIs = 56), 1 week 15 mg/kg AZA (*N* = 3, *n* = 11, ROIs = 58), 4 weeks 1.5 mg/kg AZA (*N* = 4, *n* = 10, ROIs = 74), 4 weeks 15 mg/kg AZA (*N* = 3, *n* = 10, ROIs = 57). In experiments **D–F** 1 week physiologic saline (*N* = 4, *n* = 21, ROIs = 121), 1.5 mg/kg AZA (*N* = 3, *n* = 13, ROIs = 89), 1.5 mg/kg 6-MP (*N* = 3, *n* = 16, ROIs = 104) or 1.5 mg/kg 6-TG (*N* = 3, *n* = 17, ROIs = 90). **G** Animals treated for 1 week either with saline (*N* = 13), 1.5 mg/kg AZA (*N* = 12), or 6-MP (*N* = 9) were anesthetized with ketamine-xylazine and the secretin stimulated in vivo pancreatic juice secretion was measured by cannulation of the main pancreatic duct and quantified as μL/body weight (g)/hour. The image depicts the cannulation and clipping of the main pancreatic duct during the operation procedure. The data are shown as mean ± SD, **P < 0.01, ***P < 0.001, one-way ANOVA with Sidak’s multiple comparisons tests. 6-MP = 6-mercaptopurine, 6-TG = 6-thioguanine

To investigate, whether the ex vivo observed effects could also be seen in a clinically more relevant experimental setup, we treated mice orally in vivo both with therapeutic (1.5 mg/kgbw/day) [42] and non-lethal toxic (15 mg/kgbw/day) [25] AZA doses for one week. To gain information about the exocrine pancreatic HCO_3_^–^ secretion, we isolated pancreatic ductal fragments and conducted the above-described fluorescent measurements. Our results demonstrated that one-week *per os* AZA treatment, either with therapeutic or toxic doses, significantly impaired the base fluxes of both recoveries (i.e., from alkaline or acid loads) compared to untreated control mice (Fig. 4A-C), indicating that the inhibitory effect of AZA is present after in vivo treatment as well. In addition, we also treated animals with the other two thiopurines for 1 week with 1.5 mg/kgbw daily doses. The one-week-long treatment with 6-MP and 6-TG both recapitulated the effects of the AZA treatment and significantly decreased the base flux values during recovery both from alkaline, as well as from acid loads (Fig. 4D-F). The magnitude of inhibition was not significantly different among the three thiopurines (Fig. 4E, F). This indicates that the inhibitory effect of thiopurines is not dependent on the specific conformational differences between the drugs, but it is rather a common property of all thiopurines. To assess whether the thiopurine-induced decrease of the ductal HCO_3_^–^ secretion diminishes the pancreatic fluid secretion, we measured the total pancreatic juice secretion in vivo in anesthetized mice. After 1 week of *per os* treatment with either 1.5 mg/kgbw/day AZA or 6-MP, the secretion rates were significantly reduced in both cases compared to sham controls (Fig. 4/G). Our results demonstrate that AZA and its metabolites inhibit the pancreatic HCO_3_^–^ and fluid secretion in vivo.

### AZA decreases the apical CFTR expression and CFTR-mediated Cl^–^ secretion in pancreatic ductal cells

**Fig. 5:**
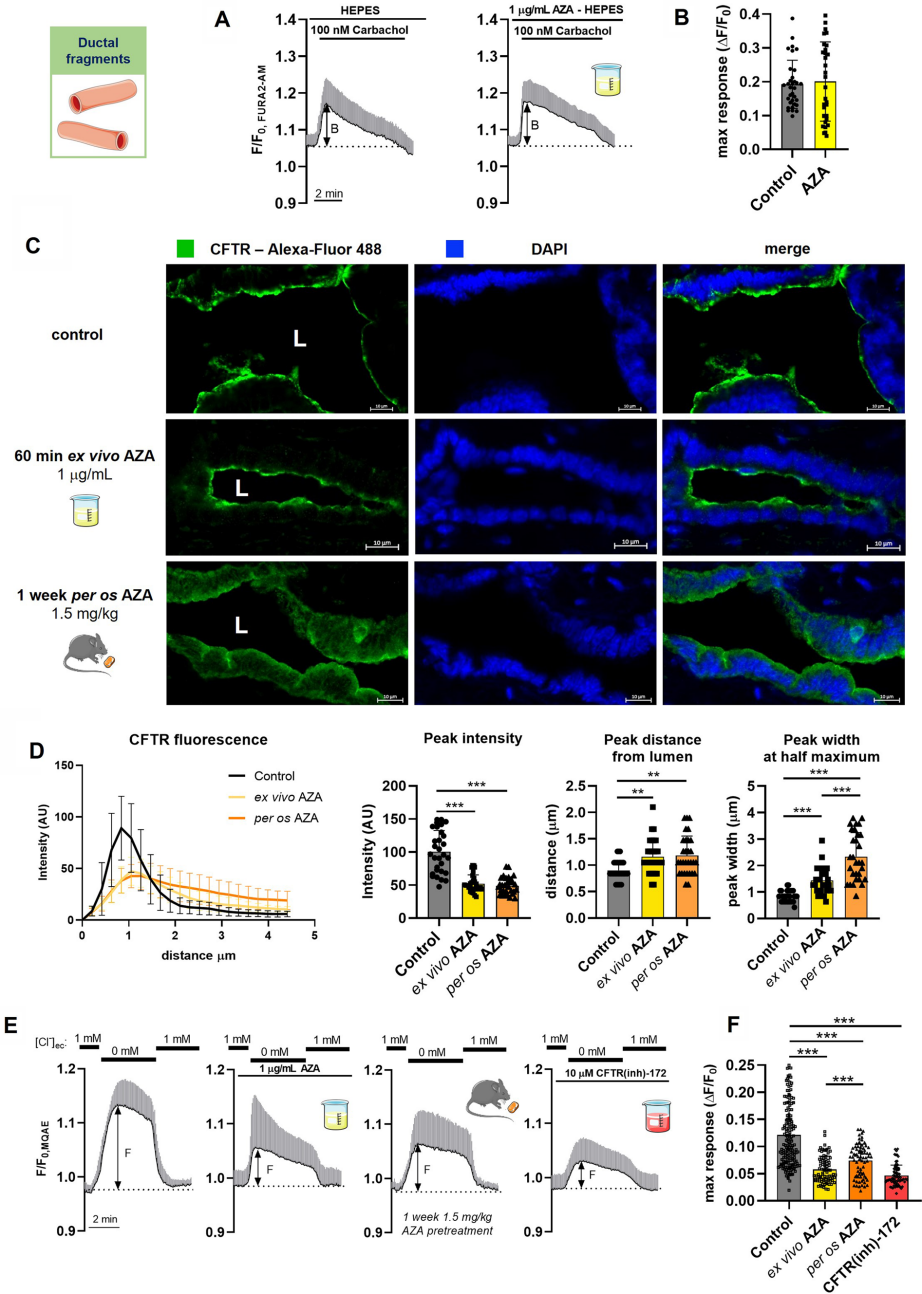
Azathioprine impairs pancreatic ductal CFTR expression and Cl^–^ secretion. **A**, **B** For the measurement of ductal Ca^2+^ signals, ductal fragments from untreated animals were isolated and stimulated with 100 nM Carbachol with a prior ex vivo perfusion with 1 μg/mL azathioprine (AZA) and without (controls). **A** Average traces of normalized FURA2 fluorescence intensity during carbachol stimulation are shown. The double-sided arrows mark how the maximal response values plotted on the B panel were calculated. **B** The magnitude of the maximal Ca^2+^ response to carbachol stimulation. 33 ROIs on 10 ductal fragments, isolated from 3 animals were used per group. The data are shown as mean ± SD, *P* = 0.073, unpaired t-test. **C**, **D** Ductal fragments isolated from untreated mice were either incubated ex vivo for 60 min with 1 μg/mL AZA, as well as controls, and ductal fragments from *per os* AZA-treated mice (1 week, 1.5 mg/kg) were incubated for 60 min in normal culture media. CFTR was labeled with fluorescent antibodies and images were captured with an LSM880 confocal microscope using a 40× oil immersion objective. **C** Representative images of immunofluorescent staining of CFTR on mouse pancreatic ductal fragments. The white “L” marks the luminal cavity. **D** Average intensity profiles of luminal CFTR fluorescence, average peak fluorescence intensity, average peak distance from the lumen, and average peak width at half-maximal intensity (N = 5 ducts per group, n = 30 profiles). The data are shown as mean ± SD, **P < 0.01, ***P < 0.001, one-way ANOVA with Sidak’s multiple comparisons tests. **E**, **F** Ductal fragments from untreated mice were perfused ex vivo either with 1 μg/mL AZA or with 10 µM CFTR(inh)-172. The Cl^–^ secretion was measured upon the withdrawal of extracellular Cl^–^ by determining the amplitude of change in the normalized MQAE fluorescence intensity. Similarly, ex vivo Cl^–^ secretion was also measured on ductal fragments isolated from animals treated *per os* for 1 week with 1.5 mg/kg AZA. **E** The average traces of normalized MQAE fluorescence intensity (± SD) during extracellular Cl^–^ depletion are shown. The double-sided arrows mark how the maximal response values plotted on the F panel were calculated. **F** Magnitude of Cl^–^ response to Cl^–^ depletion measured in different conditions. The number of animals (*N*), ductal fragments (*n*), and regions of interest (ROIs) used in the experiments: controls (*N* = 4, *n* = 19, ROIs = 174) 1 μg/mL AZA (*N* = 4, *n* = 15, ROIs = 83), 1 week 1.5 mg/kg AZA (*N* = 4, *n* = 13, ROIs = 75) 10 mM CFTR(inh)-172 (*N* = 3, *n* = 12, ROIs = 72). The data are shown as mean ± SD, ***P < 0.001, one-way ANOVA with Sidak’s multiple comparisons tests

Previously we demonstrated that pancreatitis-inducing agents, such as bile acids or non-oxidative ethanol metabolites, inhibit the pancreatic ductal HCO_3_^–^ secretion via sustained intracellular Ca^2+^ elevation [21, 43]. To test whether this mechanism plays a role in the inhibitory effect of AZA, we assessed the intracellular Ca^2+^ homeostasis of pancreatic ductal cells in the presence of AZA. Similarly to acinar cells, we could observe no significant changes either in the baseline [Ca^2+^]_i_ levels during the 10-min perfusion with 1 μg/mL AZA, or in the maximal response to 100 nM carbachol stimulation after the AZA perfusion, compared to controls (Fig. 5A, B), suggesting that AZA does not have a major influence on ductal Ca^2+^ homeostasis. On the other hand, in alcohol-induced AP, a marked reduction of the apical CFTR expression and function is observed as a highlight of the toxic effect of ethanol on the ductal cells [21]. To test whether the inhibitory effects of AZA on HCO_3_^–^ secretion are caused by disturbed CFTR expression or function, we analyzed the intracellular localization of CFTR with immunofluorescent staining. Our results showed that both incubation of ductal fragments with 1 μg/mL of AZA for 60 min ex vivo, or 1 week of *per os* treatment with 1.5 mg/kgbw AZA significantly diminished the predominantly apical staining of CFTR and triggered the retention of the protein into the cytosol (Fig. 5C, D). Then, we tested whether the disturbed apical CFTR expression also pairs with impaired Cl^−^ secretion. To investigate this, we examined the CFTR-dependent Cl^**–**^ secretion transport of the ductal epithelia. In these experiments, both the 10 min acute exposure of the ductal fragments to 1 μg/mL AZA, as well as the 1-week-long *per os* treatment with 1.5 mg/kgbw AZA impaired the luminal Cl^**–**^ efflux significantly compared to untreated controls. Importantly, the selective CFTR inhibitor CFTR(inh)-172 caused a similar inhibition of the CFTR-mediated Cl^–^ transport to that caused by AZA (Fig. 5E, F). Notably, our results revealed that AZA treatment diminishes the apical CFTR expression and significantly impairs the function of CFTR in pancreatic ductal cells.

### AZA disrupts CFTR plasma membrane retention in PDEC by inhibiting RAC1 and ezrin in mice

**Fig. 6:**
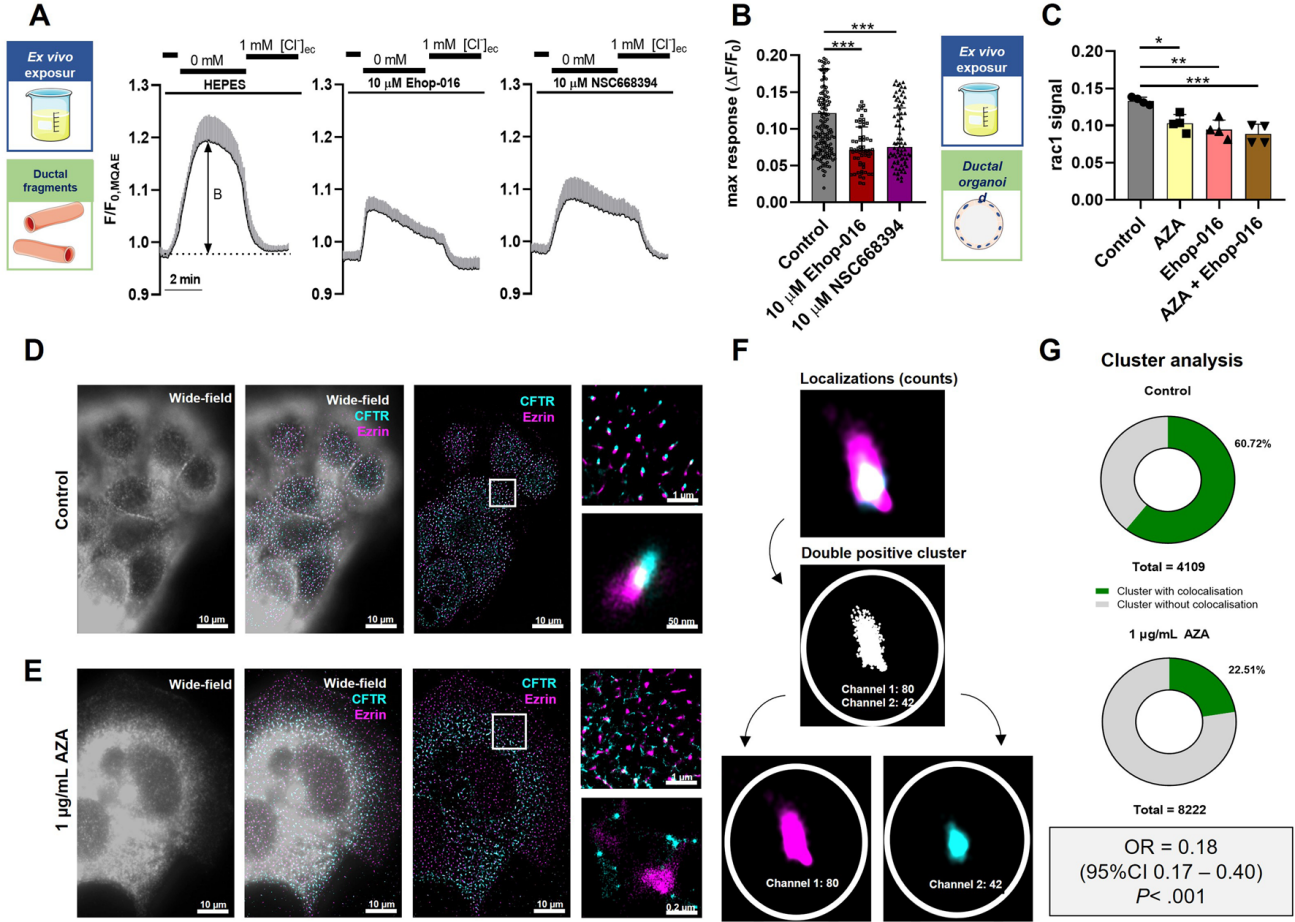
Azathioprine inhibits RAC1 activity and alters CFTR-ezrin colocalization in mouse pancreatic organoids. **A-B**, Ductal fragments from untreated mice were perfused ex vivo either with 10 µM RAC1 inhibitor (Ehop-016) or 10 µM ezrin inhibitor (NSC668394). The Cl^–^ secretion was measured upon the withdrawal of extracellular Cl^–^ by determining the amplitude of change in the normalized MQAE fluorescence intensity. **A** The average traces of normalized MQAE fluorescence intensity (± SD) during extracellular Cl^–^ depletion are shown. The double-sided arrows mark how the maximal response values plotted on the B panel were calculated. **B** Magnitude of Cl^–^ response to Cl^–^ depletion measured in different conditions. The number of animals (*N*), ductal fragments (*n*), and regions of interest (ROIs) used in the experiments: controls (*N* = 4, *n* = 19, ROIs = 174) 10 µM Ehop-016 (*N* = 3, *n* = 9, ROIs = 59), or 10 µM NSC668394 (*N* = 3, *n* = 11, ROIs = 78). The data are shown as mean ± SD, ***P < 0.001, with Sidak’s multiple comparisons tests. **C** Mouse pancreatic ductal organoids digested into single cells were incubated in vitro with either 1 μg/mL azathioprine (AZA), 10 µM RAC1 inhibitor (Ehop-016), or both for 60 min. Then cells were lysed on ice and RAC1 activity was measured with a G-LISA assay following the manufacturer’s protocol. RAC1 activity signals were measured with the G-LSIA assay (*n* = 4 per group, from 2 different cultures). The data are shown as mean ± SD, *P < 0.05, **P < 0.01, ***P < 0.001, one-way ANOVA test on all pairwise combinations. **D-G** Another set of organoids was incubated for 60 min with normal culture media (**D**) or 1 μg/mL AZA (**E**). CFTR and ezrin were labeled with fluorescent antibodies and visualized on direct stochastic optical reconstruction microscopy (dSTORM). Representative dSTORM images of CFTR-ezrin clusters in control and AZA-treated organoids show non-panel (**D**, **E)** respectively. **F** Schematic diagram of the cluster analysis process. A channel-independent cluster analysis was performed based on the collected and individually recorded blinking events. Clusters were defined using a diameter of 120 nm from the center. The number of homogeneous and double-positive clusters was determined by examining the composition of individual clusters. (In the figure, the number after the channel name for the example cluster indicates the recorded blinking events of the fluorophores. **G** The results of a cluster analysis of CFTR-ezrin colocalization. OR and 95% CI are calculated with the chi-square test, *P* < 0.001

Finally, we wanted to understand the mechanism of the AZA-induced decrease in CFTR plasma membrane localization and function. It is well-described that CFTR is tethered to the apical plasma membrane by a scaffolding protein complex, comprising Na^+^/H^+^ exchanger regulating factor (NHERF) family members and the actin cytoskeleton adaptor protein, ezrin [44–46]. Ezrin was also shown to play a role in the Protein-kinase-A-mediated regulation of CFTR [47]. Previously, AZA was suggested to inhibit RAC1 [35, 48], an activator of ezrin [44], which may play a role in the effects of AZA in ductal cells. To investigate the role of RAC1 and ezrin in the AZA-induced CFTR dysfunction, we first administered RAC1 (Ehop-016), and ezrin (NSC668394) inhibitors for 10 min to isolated ductal fragments, and measured the Cl^**–**^-efflux. Interestingly, both ezrin and RAC1 inhibitors recapitulated the effect of AZA on ductal Cl^**–**^ secretion (Fig. 6A, B). To further validate our hypothesis, mouse pancreatic organoids were used to measure RAC1 activity in ductal cells. As organoids contain epithelial cells only, no contamination of other cell types is expected [29], which may disturb RAC1 activity measurement. For this assay, the organoids were digested into single cells as previously described [23] and were incubated with 1 μg/mL AZA for 60 min, which significantly decreased the amount of active RAC1 in a G-LISA assay (Fig. 6C). Notably, the RAC1 inhibitor Ehop-016 was not able to further decrease the activity of RAC1 in the presence of AZA. Finally, we used super-resolution direct stochastic optical reconstruction microscopy (dSTORM) to quantify the colocalization of CFTR and ezrin in adherent primary epithelial cells derived from mouse pancreatic organoids. The STORM images clearly demonstrated that AZA treatment significantly decreased the number of colocalizing ezrin-CFTR clusters compared to the control cells (Fig. 6D). To quantify this decrease, cluster analysis was performed, as shown (Fig. 6E). First, the total number of localizations, then the number of the double-positive clusters was determined and given as the % of the total cluster numbers. The performed cluster analysis confirmed that incubation of the cells with 1 μg/mL AZA for 60 min markedly decreased the colocalizing probability (OR = 0.18; 95% CI 0.17 – 0.40; *P* < 0.001) of ezrin and CFTR compared to the control (Fig. 6F). To confirm these results, subcellular fractionation and western blot for ezrin and CFTR were performed. These results suggest that the level of ezrin is impaired in the cytoskeletal fraction, whereas the level of CFTR is decreased in the membrane and cytosolic fraction, which supports our observation with the dSTORM (Suppl. Figure 2). These results confirm that the impaired plasma membrane localization of CFTR in the AZA-treated ductal cells is caused by the inhibition of RAC1, which disturbs the CFTR-ezrin interaction leading to decreased plasma membrane tethering and loss of CFTR expression, which consequently impairs the CFTR function.

### Discussion

In this study, we used different ex vivo and in vivo model systems and explored the pathomechanism of TIP, which is one of the most common side effects of AZA treatment among IBD patients. Our results showed that in vivo AZA treatment increases tissue damage in the early phase of the cerulein-induced AP in mice. AZA and its metabolites impaired the expression of CFTR in the apical plasma membrane of pancreatic ductal cells via RAC1 inhibition and disruption of CFTR-ezrin interaction, leading to a consequent decrease in the exocrine pancreatic ion and fluid secretion. Notably, AZA had no primary effect on the pancreatic acinar cells.

The spectrum of adverse events during thiopurine treatment is wide, ranging from digestive intolerance, alopecia, leukopenia, and hepatotoxicity to TIP [10, 11]. Several studies, mostly with a clinical approach, tried to investigate the pathophysiology of TIP and suggested immune-mediated and genetics-associated mechanisms [15, 49]. They also mention the potential role of direct toxic mechanisms but the molecular background remains unexplored [15]. Clinical studies usually report TIP during the first 30 days of treatment [14, 41, 50]. In our study, we demonstrated that daily administration of AZA for a week has no visible effect on the exocrine pancreatic morphology in mice. On the other hand, when the mice were challenged with cerulein, the tissue damage in the early phase of the AP was significantly increased in the AZA-treated group suggesting that AZA treatment may have direct effects on the exocrine pancreas. Eventually, this phenomenon may be influenced by the immunosuppressant effect of thiopurines as well. Previously, Kerstein et al. reported amelioration of experimental pancreatitis in dogs by administration of AZA [51]. Nevertheless, our data suggest that AZA treatment increases the exocrine pancreatic damage in the presence of other stimuli that may underlie the clinically observed features of TIP.

Next, we wanted to assess the mechanism leading to the increased damage of the cerulein-stimulated exocrine pancreas. The most common pancreatitis-inducing agents, such as ethanol and bile acids, affect the pancreatic acinar [52] and ductal cells [21]. Ethanol and non-oxidative ethanol metabolites and bile acids were previously described to trigger sustained intracellular Ca^2+^ elevation and mitochondrial damage in pancreatic acinar and ductal cells, leading to intra-acinar trypsinogen autoactivation and impaired ductal secretion, respectively [21, 43]. Moreover, similar intracellular events were described in the case of Asparaginase-induced AP [6]. Previously, Foitzik et al. showed that treatment either with supra-clinical doses of AZA (10 mg/kgbw/day) or different doses of cyclosporine A, administered 6 h after the AP-induction, caused more extensive acinar cell necrosis in rats [53]. In addition, Geiger et al. showed that incubation with the toxic concentrations (5 and 10 μg/mL, respectively) of AZA elevated the intracellular Ca^2+^ level and phosphatidylserine exposure of human erythrocytes, but not with the clinically more relevant 2 μg/mL AZA concentration [38]. In our experiments, AZA had no detectable effect on the viability of pancreatic acinar cells when used in a clinically relevant concentration range. Accordingly, no alterations were registered in the intracellular Ca^2+^ homeostasis or amylase release of pancreatic acinar cells. These data suggest that in contrast to the previous findings, AZA has no visible effect on pancreatic acinar cells in clinically relevant concentrations. This aligns well with the clinical observation that the majority of AP cases during thiopurine treatment are mild to moderate, whereas severe cases are rarely reported [54].

Another possible target of pancreatitis-inducing factors are the pancreatic ductal epithelial cells [36, 37], which secrete the HCO_3_^–^-rich, alkaline pancreatic juice that washes the digestive enzymes out from the pancreatic tree thus preventing premature trypsinogen autoactivation [55]. The HCO_3_^–^ secretion is mediated by the interplay of the basolateral Na^+^-HCO_3_^–^ co-transporters (NBCe1-B), the luminal Cl^–^/HCO_3_^–^ exchangers (SLC26A3,6), CFTR channels, and the ubiquitous Na^+^/H^+^ exchanger proteins (NHE1-4) [29, 39, 56]. Of these proteins, CFTR seems to play a central role in pancreatic diseases [57, 58]. Our group has demonstrated previously that ethanol and fatty acid treatment directly reduced the expression and activity of CFTR, impaired ductal HCO_3_^–^ secretion, and led to increased severity of experimental AP in mice [21]. Also, genetic deletion of Na^+^/H^+^ exchanger regulatory factor-1 (NHERF1) – a member of a protein complex tethering CFTR to the apical membrane [45] – has also been shown to cause CFTR mislocalization in PDEC and decreased ductal secretory activity leading to a more severe experimental AP [39]. Early studies of the effect of AZA on canine exocrine pancreatic secretion from the 1980s provided controversial results. In a preliminary study, Dreiling et al. found that co-administration of intravenous AZA (100 mg) and hydrocortisone (1 mg) significantly decreased the pancreatic juice flow in dogs [59], while Broe et al. reported that infusions with both 0,5 and 5 mg/kgbw AZA increased the pancreatic flow and HCO_3_^**–**^ output in ex vivo canine pancreas preparations [60]. In our study, we provided clear evidence that both ex vivo and in vivo thiopurine treatment (AZA and its metabolites) decreases pancreatic ductal HCO_3_^**–**^ and fluid secretion in mice, in clinically relevant concentrations. Notably, for the in vivo administration, we used oral gavage feeding that completely recapitulates the pharmacokinetics of the usual clinical (oral) administration of AZA. Here, we also showed that thiopurine treatment impaired the basolateral (i.e., NHE and NBCe1) and apical ion transport activities, respectively. Previously, Bhandaru et al. found no effect of AZA on basolateral NHE in dendritic cells when AZA was applied in 2.77 μg/mL. This may suggest that the impaired basolateral transport activities may be secondary due to the damaged apical transport processes, however, we did not explore this phenomenon further in the current study.

To further characterize the inhibition of HCO_3_^–^ secretion caused by thiopurines, we analyzed the apical CFTR expression and activity of pancreatic ductal cells in the presence of AZA. CFTR channels can be found almost exclusively on the apical membrane of pancreatic ductal cells [56]. The CFTR-mediated Cl^–^ and HCO_3_^–^ transport represents a rate-limiting step in epithelial anion secretion, therefore, controlling transepithelial fluid secretion and ultimately the hydration of the epithelial luminal surfaces [61]. Several mutations of the CFTR gene lead to impaired ion channel function and consequently impaired epithelial fluid transport in organs such as the lung and the pancreas. While homozygous carriers of CFTR mutations generally develop cystic fibrosis, heterozygous carriers do not necessarily; however, they often exhibit an increased risk for pancreatitis and associated pancreatic damage characterized by elevated mucus levels, fibrosis, and cyst formation [61]. Furthermore, recent studies demonstrated that not only genetic mutations but other pancreatitis-causing insults, such as alcohol, smoking, or bile acids, can inhibit CFTR-dependent epithelial functions. Here, we also demonstrated that AZA exposure decreases the apical plasma membrane expression of CFTR, moreover the Cl^–^ secretion of ductal fragments was also significantly impaired by AZA, applied either ex vivo or in vivo. These results suggest that AZA treatment consequently diminishes CFTR function as well due to the impaired channel expression.

Finally, we wanted to clarify the mechanism of action through which AZA inhibits CFTR activity and expression. Multiple studies showed, that the inhibited ductal secretory functions and CFTR activity are at least partly a result of toxic Ca^2+^ overload in ductal epithelial cells [21, 22, 43, 62]. However, similar to acinar cells, we could find no altered Ca^2+^ signals in PDEC during AZA treatment, suggesting a Ca^2+^-independent inhibitory effect of AZA. CFTR is a member of a large plasma membrane protein complex consisting of several proteins [63]. The apical localization of CFTR in secretory epithelial cells is maintained by a scaffolding protein complex comprised of CFTR, NHERF1, ezrin, and actin [39, 64]. Ezrin is regulated by RAC1 [44], and stimulation of RAC1 has been shown to restore F508del-CFTR expression and functions in human bronchial epithelial cells [64]. Furthermore, in a previous study, AZA was suggested to inhibit RAC1 in T lymphocytes [20]. Here, we demonstrated that AZA not only inhibits RAC1 activity in PDEC but leads to impaired colocalization of ezrin and CFTR, which eventually affects the plasma membrane distribution of CFTR in mouse ductal fragments. Importantly, this observation provides a detailed molecular mechanism that could explain the impaired pancreatic ion and fluid secretion observed in AZA-treated animals.

To our knowledge, this is the first study that describes the effect of AZA on the exocrine pancreas applied in a clinically relevant setting. Although the impaired apical CFTR expression and activity observed in pancreatic ductal cells could potentially contribute to the increased sensitivity of the exocrine pancreas to other stress factors, so far clinical studies mostly highlighted only the immunologic and genetic mechanisms of TIP [49, 65]. Other serious adverse events of thiopurine usage, such as myelosuppression, are associated with thiopurine methyltransferase polymorphisms, but such an association has not been shown in the case of TIP [12]. Heap et al. identified a strong association between Class II HLA variants and susceptibility to TIP in IBD patients [49], which is in support of immune-mediated mechanisms. Another interesting finding is that TIP is observed in a higher percentage of IBD patients – especially in Crohn’s disease – than in any other patients treated with AZA [50, 54, 66]. Although these studies are supporting that immunologic processes would play an important role in TIP, exact mechanisms behind this hypothesis are not yet found, while the direct effects of AZA on CFTR activity presented in our study gives the first mechanistic insight into the pathogenesis of TIP which may open new directions in the prevention of this important adverse event. Recently, personalized evaluation of thiopurines based on pharmacogenetics in a recent study was successful to prevent myelotoxicity; however, it failed in the prevention of TIP [67]. CFTR functions, however, can be easily assessed in rectal biopsies and rectal organoid cultures offering personalized treatment options in patients with cystic fibrosis [68]. Similar approaches might prove useful in the prevention of TIP to screen patients with decreased CFTR activity, given that colonoscopy is routinely performed in the clinical follow-up of IBD patients. Furthermore, cystic fibrosis drives intense research to identify novel drugs to modify CFTR activity [69], which also holds the potential to be utilized in the prevention of TIP.

Taken together, in mice, we explored the mechanism of action of AZA on the exocrine pancreas, which exclusively affected the secretory activity of pancreatic ductal cells. The RAC1 inhibition by thiopurines led to an impaired ezrin-CFTR interaction and disturbed CFTR expression on the apical plasma membrane of the pancreatic ductal cells. Our observations may be relevant not only in the pancreas but in other secretory organs as well.

### Supplementary Information

Below is the link to the electronic supplementary material.

Supplementary file1 (DOCX 1252 KB)

## Data Availability

The datasets generated during and/or analyzed during the current study are available from the corresponding author on request.

## Abbreviations

6-MP: 6-Mercaptopurine
6-TG: 6-Thioguanine
6-TGNs: 6-Thioguanine-nucleotides
AZA: Azathioprine
AP: Acute pancreatitis
AU: Arbitrary unit
kgbw: Body weight in kilograms
BCECF-AM: 2',7'-bis-(2-carboxyethyl)-5-(and-6)-carboxyfluorescein, acetoxymethyl ester
[Ca^2+^]_i_: Intracellular Ca^2+^ level
CFTR: Cystic fibrosis transmembrane conductance regulator
[Cl^–^]_i_: Intracellular Cl^–^ level
DIAP: Drug-induced acute pancreatitis
dSTORM: Direct stochastic optical reconstruction microscopy
EPS: 4,6-Ethylidene(G1)-4-nitrophenyl(G7)-a-(1–4)-D-maltoheptaoside
FURA2-AM: Fura-2-acetoxymethyl ester
IBD: Inflammatory bowel disease
IP: Intraperitoneal
MQAE: N-(Ethoxycarbonylmethyl)-6-methoxyquinolinium bromide
NHE: Na^+^/H^+^ exchanger
NHERF1: Na^+^/H^+^ exchanger regulatory factor-1, SLC9A3R1
NBC: Na^+^-HCO_3_^–^co-transporters
PDEC: Pancreatic ductal epithelial cell
pH_i_: Intracellular pH
RAC1: Ras-related C3 botulinum toxin substrate
SLC26: Solute carrier family 26
TIP: Thiopurine-induced acute pancreatitis
