# Supplementary material for: Thiopurines impair the apical plasma membrane expression of CFTR in pancreatic ductal cells via RAC1 inhibition
Source: Cell Mol Life Sci. 2023 Jan 7;80(1):31. doi: 10.1007/s00018-022-04662-y (PMC9825359; doi:10.1007/s00018-022-04662-y)
Supplement: Supplementary file 1 — Supplementary file1 (DOCX 1252 KB) [file 18_2022_4662_MOESM1_ESM.docx]

**Supplementary methods**

**Ethanol-induced acute pancreatitis**

For the induction of ethanol + fatty-acid pancreatitis, we used an adaptation of the method originally developed by Huang et al (Huang *et al.*, 2014). Briefly, mice were pre-teated either with daily doses of 1.5 mg/body weight (BW) kg Azathioprine (AZA) or physiologic saline (PS) as sham controls for 1 week. Then on the 7^th^ day of treatment, mice received intraperitoneal injections administered hourly, either a total of two ethanol injections mixed with palmitoleic acid (EtOH+POA; 1.35 g+150 mg/BWkg) or a total of two PS injections. Twenty-four hours after the first injection, the mice were sacrificed, blood samples were collected by cardiac puncture, then pancreata were removed, washed with physiologic saline, cleaned from lymph nodes and fat, weighed, and stored at +4°C in 4% formaldehyde.

**Subcellular fractionation assay and Western blot**

Mouse pancreatic duct organoids were grown and collected as described before (Madácsy, Cell Mol Life Sci. 2022 Apr 28;79(5):265). The collected organoids were divided into two equal parts consisting of a control group (CTR) and an AZA-treated group (AZA, 1 µM, 30 min). Following treatment, a cytoplasmic-, a membrane-, a nuclear-, and a cytoskeletal extract fraction was isolated from the organoids with a Subcellular Protein Fraction Kit according to the manufacturer’s guidelines (Thermo, 78840). Protein concentration was measured with a BCA protein Assay Kit according to the manufacturer’s guidelines (Thermo, 23225). Equal amounts of total protein of all fractions were separated with SDS-PAGE and blotted on PVDF membranes. To identify the protein targets, the following antibody combinations were used: ezrin, mouse anti-ezrin antibody (Abcam, ab270525) followed by goat anti-mouse antibody coupled to HRP; CFTR, rabbit anti-CFTR antibody (Alomon Labs ACL-006) followed by goat anti-rabbit antibody coupled to HRP (Thermo, 31460). Next, resulting signals were visualized with enhanced chemiluminescence according to the manufacturer’s guidelines (BioRad, 170-5060). The density of captured chemiluminescence was analyzed from images with the Gel Analyzer plugin integrated into FIJI Software (NIH, version 1.53c).

## Supplementary Figure 1.

##
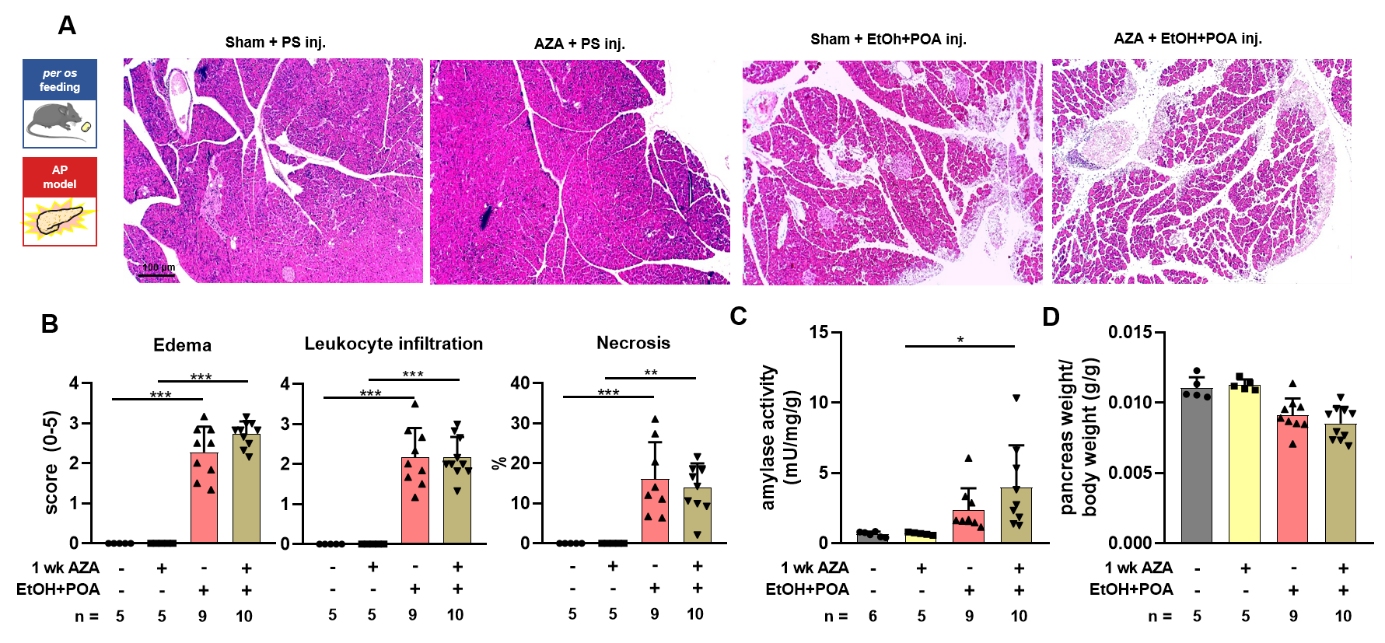


**A**, Representative images of hematoxylin-eosin (H-E) stained slides of formaldehyde-fixed pancreata captured with 50× total magnifications and corrected for background illumination. Images were captured with a Zeiss Axio Imager.M2 microscope. **B,** Histology scores and percentage of necrosis of H-E slides in EtOH+POA induced acute pancreatitis. Each slide was analyzed in at least 3 different fields of view, by three independent observers. (*n = 5, sham + PS; n =5, AZA+ PS; n= 9, sham + EtOH+POA; n =10, AZA + EtOH+POA groups.*). **C**, Activity of serum amylase in EtOH+POA induced acute pancreatitis. **D**, Pancreas weights of mice normalized to body weight in EtOH+POA induced acute pancreatitis. Scale bar: 100 µm. The data are shown as mean ± SD*,* **P*< 0.05, ****P* < .001, one-way ANOVA with Sidak’s multiple comparisons tests.

##
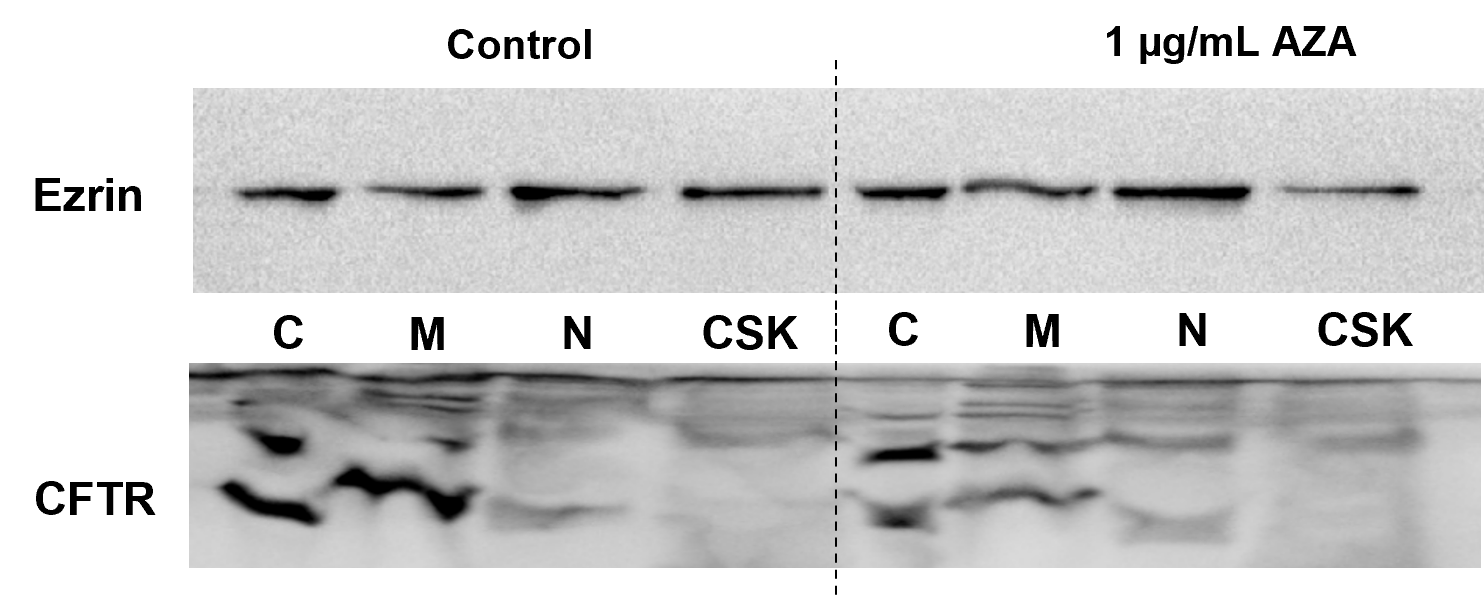


## Supplementary Figure 2.

## A, Images of Western blots after subcellular fractionation assay. The subcellular fraction are denoted with the following abbreviations: C = cytoplasm, M = membrane, N = nucleus, CSK = cytoskeleton.
